# Maintaining normality when serving a prison sentence in the digital society

**DOI:** 10.3325/cmj.2018.59.335

**Published:** 2018-12

**Authors:** Eva Marie Toreld, Kristin Opaas Haugli, Anna Lydia Svalastog

**Affiliations:** 1 Østfold University College, Department of Health and Social Studies, Fredrikstad, Norway; 2 Oslo Metropolitan University, PhD Program Social Work and Social Policy, Oslo, Norway *marie.toreld@hiof.no*; 3 Norwegian Correctional Services, Kongsvinger Prison, Kongsvinger, Norway

## Digital exclusion and social marginalization, new challenges for health and well-being

A just and well-functioning correctional service is a key institution in the modern democratic society (1). It affects the safety, health, and well-being of individuals, groups, and society at large. In this article, we will discuss how the digital society has created new conditions for society, and how the correctional services need to understand and meet these conditions. The digital society has made digital communication a vital part of everyday life (2). We communicate, seek and get information, apply for work or welfare benefits, and manage our daily tasks through digital technologies and services. Although internet access is global, the distribution of digital technologies varies. As society has become digital, new divides have been created. Some people are continuously connected to the internet, while some are rarely online. Studies of these divides have shed light on how individuals and groups, in particular elderly people, struggle for digital participation and risk social exclusion (3,4).

In this article, we focus on an important segment of society, people sentenced to imprisonment. The digital society has not so far been an explicit theme in studies of normality and normalization in the correctional services. Therefore, our aim is to address the exclusion from digital technology during imprisonment in relation to the principle of normality and normalization. This exclusion can make active participation in society difficult, lead to social exclusion, and undermine social relations during and after serving a sentence.

The Knowledge Landscapes network initiated investigations and discussions on how people search for health-related knowledge in the digital society. In the *Croatian Medical Journal* stream of Knowledge Landscapes articles, the concept of Knowledge Landscapes has been defined to help us understand health as it is constituted – understood, handled, maintained, and communicated in the digital society (5). The Knowledge Landscapes stream focuses on knowledge in the online environment as part of a multidirectional communication, and Knowledge Landscapes has been used as a key metaphor when searching for knowledge in the digital environment (6). Knowledge Landscapes has also been used as an analytical concept to describe the navigation toward knowledge important for the individual life (7). The key focus of this article is how the tentativeness of the online environment affects people who are sentenced to prison and thus are not permitted to participate in online interactions.

## Correctional services

The prison sentence in Norway entails the restriction of liberty. No other rights are removed by the sentencing court (8). This means that the prisoners keep the same rights as outside prison, eg, the right to vote, the right to health care, and the right to education. You need a reason to deny a sentenced offender his or her rights, not to grant them. The general rules are that: “No-one shall serve their sentence under stricter circumstances than necessary for security in the community” and “During serving of a sentence, life inside should resemble life outside as much as possible” (8). This corresponds well with the European Prison rules published by the Council of Europe (9): “Life in prison shall approximate as closely as possible the positive aspects of life in the community.” In the commentary on the recommendations to European prison rules this is further explained as follows:

“Rule 5 emphasises the positive expects of normalisation. Life in prison can, of course, never be the same as life in a free society. However, active steps should be taken to make conditions in prison as close to normal life as possible and to ensure that this normalisation does not lead to inhumane prison conditions” (*Rec (2006)2 of the Committee of Ministers to the member states on the European prison rules*) (9)

In Norwegian prisons, internet use is banned, except for educational purposes, and telephone use is highly restricted. At the same time, correctional services aspire to rehabilitate the offenders and prepare them for release and reintegration into society. This means a society they have not had a chance to keep up with and a society that requires knowledge or skills that they might not have mastered in the first place.

## The principle of normality and normalization in correctional services

If one of the principles of Norwegian Correctional Services is the principle of normality (4), and if life in prison should, as far as possible, resemble the society outside the prison, does exclusion from the digital environment distort the correctional services’ concept of normality? Does it lead to inmates serving their sentence under stricter circumstances than necessary? In accordance with the Norwegian Execution of Sentences Act, section three (10), the correctional services should make it possible for the convicted person to make an effort to avoid new criminal acts. How might the exclusion from digital technology affect this and the process of reintegration for prisoners? Is it possible to prevent that deprivation of liberty implies deprivation of a normal life? These questions need to be discussed in legal terms, but also in societal and ethical terms. Maybe opposite to the currently applied exclusion principle, the knowledge on how to use digital technology should be taught in prisons?

First, we need to explain what it means to maintain normality when serving a sentence in the digital society. Marianne Vollan (Director, Norwegian Correctional Services Directorate, 2013-2018) proposes three aspects of normality (11): a) medical, b) statistic, and c) cultural or moral normality. By medical normality she refers to being healthy, by statistic normality she refers to being in the middle of the curve, while by cultural or moral normality she refers to the “right” way of living by society’s standards. Inmates in Norwegian prisons experience huge challenges when it comes to health, education, housing, substance abuse, etc (12-15). It can be argued that they deviate from normality in all three aspects. This also corresponds well with the research on prisoners in other countries (16-18).

Normalization is a result of the correctional services’ activities and is related to reintegration and rehabilitation (19). Former inmates are expected to be good active citizens, without relapsing to crime, and live within the boundaries of all tree aspects of normality. However, being an active citizen in the digital society requires the use of digital skills. This means that all three aspects presented by Vollan need to be understood in relation to the digital society. Even though not all citizens have good digital skills, one deviates from the middle of the curve if he or she has weak or no skills. In Norway, citizens communicate with the administration through digital, online services (20). Being able to navigate online, to find what you need in the knowledge landscapes, has become a part of our culture and a part of being “a good citizen.”

## Digital challenges in the prison context

Knowledge about the digital society, its services, and platforms/software is tentative and in constant change. This means that the knowledge landscape related to the digital society is in constant change. Those living outside of prison can adjust to the constant changes. In prison one is not only separated from society but is also gradually alienated from these changes. The insights one might have had into how the digital society works are no longer updated or relevant

In Norway two new prisons will open in 2020. As a part of designing and developing these prisons, a digitization project is initiated. Its main objective is to develop concrete proposals for digitization that are forward-looking, contribute to streamlining work processes and tasks, and strengthen communication without removing the direct human contact between employees and inmates (21). The aim is to offer digital services to the inmates in their cells throughout the correctional services (21) in order to create digitally capable citizens.

Although the infrastructure in these new prisons will allow the extended use of modern technology, giving prisoners access to digital devices is a controversial issue. A big concern is that the prisoners will use digital technology to keep up their criminal activities when serving the sentence. Another concern is that it could create security problems in prison. In addition, the public opinion largely affects what is “politically” possible to achieve (22). What do prisoners deserve? In-cell television is no longer a big issue. However, what about other technical devices and digital access to the society outside prison? When is the right time to bring the prisoners to the technically advanced 21st century – the society they will be released to and that requires many new skills they are expected to master? Is it possible to get knowledge and navigate in the digital society without access to digital media? Are all inmates a threat to security if they get access? There will always be the risk that a minority will use their new skills for “anti-social purposes” (19,23). An interesting point is that access to digital media is not an issue in persons who serve the sentence with electronic monitoring. They have full access, like any of us. Subsequently, when an inmate is released or released on probation – there are no digital restrictions.

## Improving rehabilitation and reintegration

Keeping in touch with family while serving a sentence through in-cell-telephone or Skype could positively affect rehabilitation, resettlement (23), and keeping up normality. It could also eliminate some of the tension of prison life (22,23). Jewkes and Reisdorf (22) argue that digital exclusion during the sentence leads to long-term and deep social exclusion. The Correctional Services in Norway recognize the benefits of keeping in touch with family when long distances make it difficult for the family to visit. A directive by Correctional Services Directorate (24) regulates the inmates’ access to “video-conversations.” It states that video-conversations require technical equipment and other resources, such as staff. In other words, it is not a right of the inmates but something they can apply for in some prisons, under defined circumstances.

BRIK (*Behovs- og Ressurskartlegging I Kriminalomsorgen*; Needs and Resources Assessment Tool for the Norwegian Correctional Service), a digital assessment tool used in the correctional service in Norway, summarizes the needs and resources of the convicted offenders. This tool has not, however, focused on digital skills. To our knowledge no research has assessed digital skills of the Norwegian inmates. A study of the effect of digital technology on prisoner behavior and reoffending (23) in 13 prisons in the United Kingdom indicates prisoners' lack of digital skills. This study also shows that using digital teaching techniques can improve prisoners’ rehabilitation. McDougall et al (23) emphasize the importance of acquiring and using basic skills needed to successfully function in society. Developing digital skills helps coping in a non-criminal manner, reduces reoffending, and affects well-being (23). It reduces frustration during imprisonment, improves relationships with prison officers, family, and friends, and gives more confidence in life skills.

Introducing digital self-services for inmates, such as a desktop that they can use to administrate their shopping, account, visits, communication with social services, and other public institutions, can help them feel in more control over their lives and more confident about being part of the modern society (23). This might encourage the inmates to be more in control of their rehabilitation process, which is in accordance with the main objective of the Norwegian correctional service.

It does not have to be the question of full access or no access to digital technology in prison. However, it is necessary to acknowledge digital inequalities experienced by prisoners and accept that we all are living in a digital and technology advanced society. Society could benefit from further developing the opportunities that digital technology provides when it comes to normalization, rehabilitation, and resettlement of inmates. It is necessary to assess the inmates’ digital needs and recourses and facilitate the training of the digital skills required in the contemporary society. It is also important to address the attitude toward inmates’ access to digital technology and the consequences of denying them all access.

## Conclusion

In the digital society, serving an off-line sentence might lead to unintended consequences. The digital society is build up on new technology and software that are constantly being renewed and developed. When a person is convicted to a prison sentence, he or she is, in addition to being locked up, left on the outside of the constant changes of technology and software. An important question is whether it is possible to keep the principle of normality and aim for normalization in the 21st century by giving the prisoners access to digital technology on a larger scale than today. In addition, an off-line sentence implies a deconstruction of social skills and relations, which might undermine a person’s ability to reintegrate into society and his or her somatic, mental, and social health.

The inmates need to be able to navigate online to search for information and services; otherwise their re-integration might be at risk. Being forced to live off-line widens the gap from those who are following the digital society’s constant process of change. This might be perceived as a double punishment, and this implication is worth further research.
